# The Potential Application of Visible-Near Infrared (Vis-NIR) Hyperspectral Imaging for Classifying Typical Defective Goji Berry (*Lycium barbarum* L.)

**DOI:** 10.3390/foods13213469

**Published:** 2024-10-29

**Authors:** Danial Fatchurrahman, Federico Marini, Mojtaba Nosrati, Andrea Peruzzi, Sergio Castellano, Maria Luisa Amodio, Giancarlo Colelli

**Affiliations:** 1 Dipartimento di Scienze Agrarie, Alimenti, Risorse Naturali e Ingegneria (DAFNE), Università di Foggia, Via Napoli 25, 71122 Foggia, Italy; sergio.castellano@unifg.it (S.C.); marialuisa.amodio@unifg.it (M.L.A.); giancarlo.colelli@unifg.it (G.C.); 2 Department of Chemistry, Sapienza University of Rome, P. le Aldo Moro 5, 00185 Rome, Italy; federico.marini@uniroma1.it; 3 Biosystems Engineering Department, Shiraz University, Shiraz 71946-84471, Iran; m87.nosrati@gmail.com; 4 Dipartimento di Scienze Agrarie, Alimentari e Agro-ambientali, Università di Pisa, Via del Borghetto 80, 56124 Pisa, Italy; andrea.peruzzi@unipi.it

**Keywords:** PLS-DA, multivariate analysis, defective goji, hyperspectral imaging, non-destructive

## Abstract

Goji berry is acknowledged for its notable medicinal attributes and elevated free radical scavenger properties. Nevertheless, its susceptibility to mechanical injuries and biological disorders reduces the commercial diffusion of the fruit. A hyperspectral imaging system (HSI) was employed to identify common defects in the Vis-NIR range (400–1000 nm). The sensorial evaluation of visual appearance was used to obtain the reference measurement of defects. A supervised classification model employing PLS-DA was developed using raw and pre-processed spectra, followed by applying a covariance selection algorithm (CovSel). The classification model demonstrated superior performance in two classifications distinguishing between sound and defective fruit, achieving an accuracy and sensitivity of 94.9% and 96.9%, respectively. However, when extended to a more complex task of classifying fruit into four categories, the model exhibited reliable results with an accuracy and sensitivity of 74.5% and 77.9%, respectively. These results indicate that a method based on hyperspectral visible-NIR can be implemented for rapid and reliable methods of online quality inspection securing high-quality goji berries.

## 1. Introduction

Goji berries are recognized as modern superfruits and are widely acknowledged for their outstanding health benefits due to their high antioxidant content [1]. The fruit, derived from a plant of the Solanaceae family (*Lycium barbarum* L.) native to Asia, arrived in Europe in the 18th century due to its renowned nutritional and medical attributes [2]. Being delicate, goji berries are prone to mechanical injuries during harvest and postharvest handling [3]. A comprehensive classification of disorders in fresh goji berries has been previously reported, including fungal infection, pitting, bruises, and peel disorders [4]. In addition, goji fruit demonstrates a heightened sensitivity to the occurrence of black spots and physiological alterations related to the water conditions of the flesh rind during storage induced by water loss [5]. Finally, as for many fruits, mold incidence may limit the commerciality of goji berries, particularly in case of elevated temperatures preceding or following harvest and prolonged storage durations [6]. Due to these alteration factors, the absence of defects is a critical factor in conditioning the freshness and the commercial values of goji fruit [7]. Goji berry sorting is a labor-intensive process typically carried out by hand. The goal is to remove any defective berries. Like other manual processes, manual sorting is influenced by psychophysical conditions of the assessor, like fatigue, personal routines, as well as external factors including lighting, belt speed, and working circumstances in general. These challenges are even greater for little fruit because of their small size, implying a high number of fruit to be sorted in the time unit and reduced visibility for mild, barely perceivable defects. Moreover, the damaged part can sometimes lay on the concealed side of the conveyor belt and this makes the detection of the defects difficult, even on stationary belts [8]. To mitigate these challenges, exploring automated and alternative optical methods is necessary. Such methodologies have been demonstrated to be effective in the quality evaluation of fresh agricultural and horticultural crops in recent years [9,10,11,12,13]. The application of defect identification by using color information has already been used in various fields through machine vision systems, serving as accurate and cheap grading systems [14,15,16,17,18,19]. Nevertheless, this approach has limitations in its applicability for hidden defects and early defect recognition before symptoms manifest visibly. In this context, the merit of hyperspectral imaging (HSI) lies in its capability to capture information about internal compositional and structural alterations stemming from physiological and mechanical damages. The electromagnetic radiation generally used is related to Near Infrared (NIR) and Visible-Near Infrared (Vis-NIR) spectrum portions, acquired with two different sensors, and can predict chemical compositions in fruit without necessitating preliminary sample treatment [8]. Hyperspectral imaging (HSI) and machine learning were used to distinguish typical defects in ‘Algeri’ loquat fruit, such as purple spots, bruising, russeting, or flesh browning [5]. Furthermore, the detection of hidden bruises in kiwifruit using HIS-NIR and a parallelepipe classifier attained an overall error rate of 14.5% [20]. HIS was also used to identify imperceptible injury in the ‘Manila’ mango skin with an accuracy of 91.4% [21]. Yuan et al. [22] employed Vis-NIR hyperspectral imaging technology in conjunction with Partial Least Squares Discriminant Analysis (PLS-DA) to achieve prompt recognition of internal damage in jujube fruit. The assessment was conducted at five different temporal points following mechanical damage after 2, 4, 8, 12, and 24 h from the damage. The study revealed that the obtained model effectively identified damages in jujube fruit after 8 h from the occurrence of the bruising, demonstrating acceptable accuracy. PLS-DA is frequently employed to alleviate the elevated complexity of hyperspectral data, addressing issues like multicollinearity, and has been successfully applied for various discrimination purposes. Notably, it has been used for discriminating strawberries from three different methods of production systems [23], early detection of chilling injuries [24], and the classification of healthy, frostbitten, and diseased sweet potatoes [25]. Other authors [26] also reported the classification of goji berries’ phytochemical profile (i.e., sugar, amino acids, protein, nucleosides, and nucleobases) from five different regions. Consequently, the objective of this study was to investigate and report the application of HSI coupled with PLS-DA for the detection of different disorders of goji berries, to provide an automated, rapid, and reliable method to be implemented for online quality inspection and secure high-quality standards for the processing or distribution chain.

## 2. Materials and Methods

### 2.1. Quality Assessment

**Figure 1:**
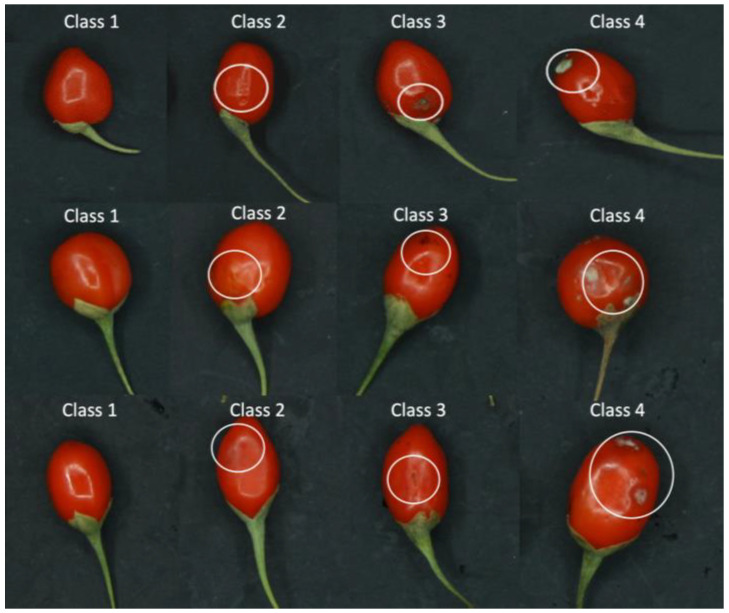
Sound goji berry fruit appearances (Class 1), mildly damaged (Class 2), moderately damaged (Class 3), and severely damaged fruit (Class 4).

Identified disorders included fungal infection, pitting, and bruises, as described in a comprehensive classification reported in a previous study [4]. A five-expert panel classified the goji berries into four distinct classes, as illustrated in Figure 1, based on the presence and severity of defects. Panelists agreed on a photographic scale with a brief description of each class to be used as a reference, following the methods previously used for a visual quality assessment of many fruit and vegetables [27]. Class 1 corresponds to healthy, undamaged fruit. Class 2 includes berries with minor defects, such as slight bruising, which are not easily visible to the naked eye. Class 3 represents berries with moderate damage, such as pitting. Class 4 comprises berries with severe damage, such as those infested with molds. The five expert panelists individually assigned the class to each fruit. A one-way ANOVA test was used to statistically verify the null hypothesis of the effect of the panelist. In the few cases where the panelist’s evaluations differed, the fruit was attributed to the class with the highest number of choice.

It is worth mentioning that bruising is commonly caused by mechanical impacts during the harvest or postharvest processes during sorting, packaging, and storage. This damage is often not apparent until after several days, when the damage is becoming more severe. The damage will often cause skin cracking during storage, so the open wound will be easily contaminated by mold. Additionally, pitting is a type of damage that may be associated with chilling injury or can be an initial sign of mold manifestation.

### 2.2. Fruit Samples

**Figure 2:**
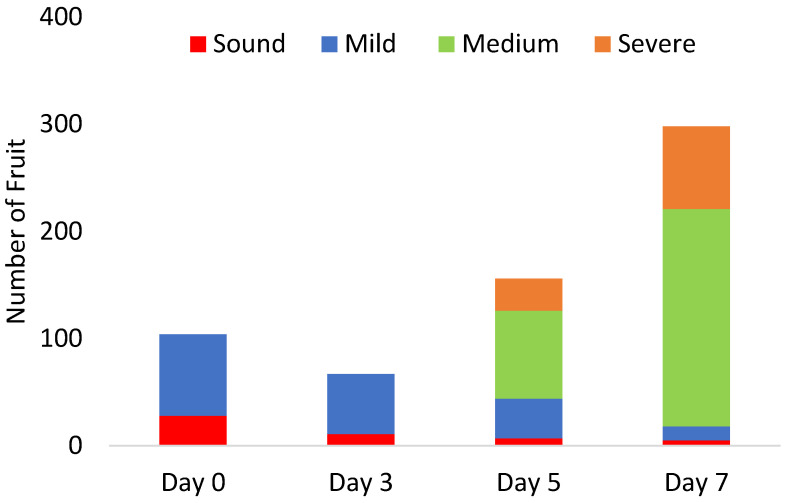
Evolution of defect incidence in goji berry fruit during storage.

In an open field (Castellaneta Province, Italy), roughly 600 g of goji fruit (*Lycium barbarum* L.; cultivar ‘sweet berry’) were collected at the commercial maturity stage on day 6 of July 2022. At harvest day, 104 fruit were scanned comprising 28 sound fruit and 76 mild-damage fruit. The remaining fruits were divided into three groups and kept at 0 °C, 5 °C, and 10 °C with a 95% relative humidity to increase the diversity of possible defects. Assessments were conducted after 3, 5, and 7 days of storage, involving a sample size of 67, 156, and 298 fruit, respectively. The evolution of defect incidence in goji berry fruit during storage is illustrated in Figure 2. Out of the total 625 fruit assessed, the distribution across these categories is sound: 51 fruit, mild damage: 182 fruit, moderate damage: 285 fruit, and severe damage: 107 fruit.

At the time of harvest, two types of defects were observed (i.e., sound and mild), which remained consistent until 3 days of storage. After 5 days of storage, more severe defects (i.e., medium and severe) were detected, with their incidence worsening by the end of the storage period.

### 2.3. Hyperspectral Image Acquisition and Spectral Extraction

Version 1.4, a hyperspectral line-scan scanner produced by DV srl, Padova, Italy, was used for the hyperspectral image acquisition and contained 5 to 10 fruit. This device integrates a camera with a Charge-Coupled Device (CCD) specifically engineered for the Vis-NIR region, possessing a spatial resolution of 27.9 pixels/mm^2^ and 5 nm spectral resolution. The spectral coverage spans the wavelength range of 400–1000 nm. To illuminate the samples, a stabilized light source as the excitation system, which consisted of a cooled halogen lamp, was set; the GigE Vision was employed as the interface, providing a field of view (FOV) of 37 degrees.

**Figure 3:**
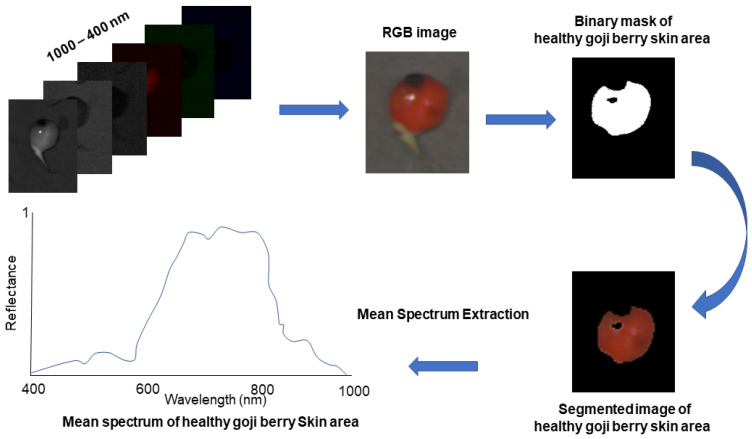
Flowchart depicting the process of hyperspectral image acquisition leading to the extraction of the mean spectrum averaged over the sound pixels of each fruit.

The extraction of pixel spectra from each fruit was performed employing a self-developed code run in MATLAB-2018b. During this process, the spectral profile of each pixel within the fruit image was extracted by excluding abnormal regions that could distort the spectral fingerprint due to glare caused by incident light. Concurrently, as depicted in Figure 3, defective regions with rapid changes in reflectance were identified. This step ensured the retention of pixels belonging to sound tissues surrounding defects. Subsequently, the spectra of these selected pixels were averaged, resulting in a single spectrum for each fruit. Based on sensorial evaluation, these spectra were labeled as either sound or defective.

### 2.4. Assessment of Chemometric

#### 2.4.1. Principal Component Analysis (PCA)

Principal Component Analysis is widely utilized in spectral data analysis as a powerful tool for feature extraction and spectra interpretation. This methodology involves transforming the input parameters into principal components (PCs), which are new sets of independent and orthogonal variables. A linear combination of the initial variables is represented by each PC, and their ranking is based on their contribution to the total variance present in the data. Generally, the initial principal components encapsulate the most pertinent information about the representative instances. The scores of these key PCs can be extracted to provide useful information for investigation purposes [28].

#### 2.4.2. Analysis of Partial Least Square-Discriminant (PLS-DA)

The PLS-DA functions as an algorithm that establishes a connection between the characteristics of the sample and spectral intensity [29]. This model works by grouping samples, converting the calibration dataset into latent variables, incorporating prior labeling of the sample classes [30]. PLS-DA is an extension of partial least squares regression (PLSR), in which regression is developed between the X and Y variables (dummy binary vector used to label the samples). This regression facilitates the model in distinguishing and classifying samples into different groups. The classification is based on the dummy matrices used for model construction, used to identify the classes [29]. In practical terms, the model’s accuracy is assessed by predicting outcomes using an external dataset comprising samples not utilized for model calibration and selection. A repeated double cross-validation (rDCV) method was employed to ensure the robustness of the model. The rDCV incorporates two nested cross-validation loops: an inner loop and an outer loop. The inner one is dedicated to tasks related to model selection, including the optimization of pre-processing methods and the identification of the appropriate number of latent variables. Top of form to prevent bias introduced by specific sample divisions into different cross-validation groups, the entire procedure is iterated 50 times. This iterative process is referred to as rDCV [31]. Multiple pre-processing methods were examined in evaluating the dataset, containing derivatives with different points and orders, the standard normal variate (SNV), and their diverse combinations. For each segment of the outer cross-validation loop, the model is optimized and calculated on a training set made up of spectra in the remaining nine segments of the outer loop. The best model was identified by taking into account latent variables, whereas the best pretreatment was accomplished by selecting the combination, leading to the minimal classification error in the inner cross-validation loop. Among the various pre-treatments explored, the most successful method for achieving the intended classification was the combination of mean centering and the second derivative (Savitzky–Golay, 15 points window, third-order polynomial).

**Figure 4:**
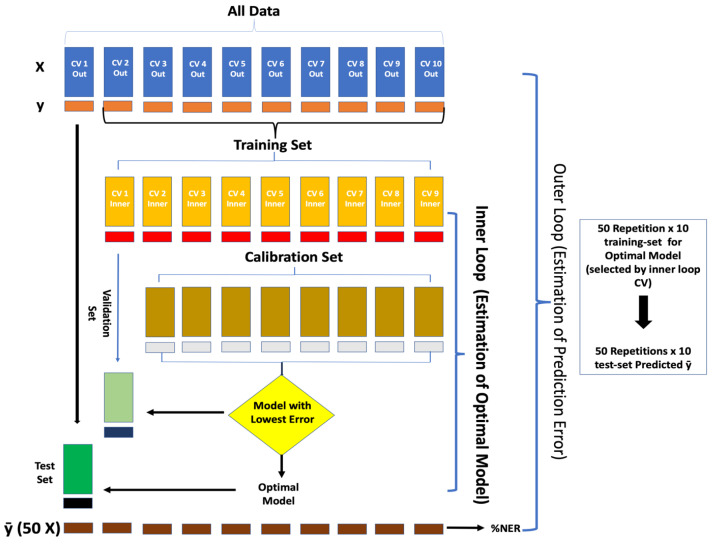
Illustration of repeated double cross-validation (rDCV) method [31].

In the case of PLS-DA for sound and defective classes, the classification strategy integrated a Covariance Selection algorithm (CovSel) for variable selection and validation through an rDCV approach. The rDCV encompassed 10 segments in the outer loop, nine segments in the inner loop (Figure 4), and as many as 50 double cross-validations (DCV) runs. Before the construction and validation of the model, the spectra underwent pre-treatment, including Multiplicative Scatter Correction (MSC), second derivative (Savitzky–Golay, 15 points window, third-order polynomial), and finally, mean centering. Every outer loop split, corresponding to every set of samples in external validation, involved the selection of optimal model complexity and the most suitable subset of original variables. These choices were made to minimize the average classification error on the samples within the inner loop and ensure the robust performance of the model. The performance in classifying the outer loop samples is considered as samples for external validation.

Furthermore, in the case of PLSDA for the four classes of damages (i.e., sound, mild, moderate, and severe), the same approach was used, aimed to estimate the severity of the defects, categorized as “sound”, “mild”, “moderate”, and “severe”. Significant pre-processing steps were implemented, encompassing Multiplicative Scatter Correction (MSC), computation of second derivative (Savitzky–Golay, 15 points window, the third-order polynomial), and mean centering. The study employed an rDCV, involving 10 outer loop segments and nine inner loop segments, and a total of 50 double cross-validation runs. 

Correspondingly, for the variable selection phase integrated into the classification strategy, a consistent subset of 34 variables was chosen in over 80% of cases. Specifically, these variables were selected in at least 400 out of the 500 computed models from 10 outer loop splits and involving 50 time runs. To assess the efficacy of this choice in more detail, a PLS-DA model was developed using only these 34 variables, and it was then confirmed using the same rDCV technique.

Furthermore, Sensitivity (Equation (1)), Specificity (Equation (2)), and Accuracy (Equation (3)) were used to assess performance in both the calibration and prediction sets.

(1) $$Sentivity\left(SENS\right)=\frac{true\;positives}{\left(true\;positives+false\;negatives\right)}$$

(2) $$Specificity\left(SPEC\right)=\frac{true\;negative}{\left(true\;negatives+false\;positives\right)}$$

(3) $$Accuraacy\left(ACC\right)=\frac{true\;positives+true\;negatives}{\left(true\;positives+true\;negatives+false\;positives+false\;negatives\right)}$$

## 3. Results and Discussion

**Figure 5:**
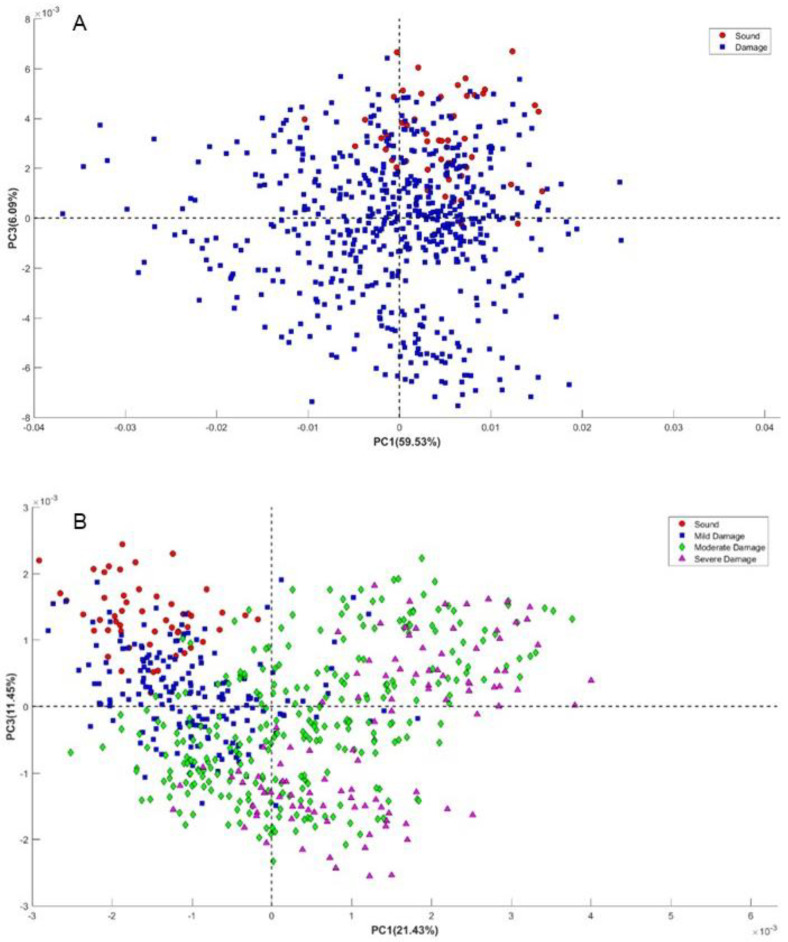
PCA score plot in two classes of sound and defective (**A**) and four classes of sound, severe, moderate, and mild damaged fruit (**B**) using pre-processed spectra.

Out of the total 625 fruit assessed, the distribution across these categories is as follows: sound: 51 fruit, mild damage: 182 fruit, moderate damage: 285 fruit, and severe damage: 107 fruit. The color image feature extracted from the segmented image shown in Figure 3 could not allow the discrimination of mild and moderate defects, for which hyperspectral imaging was needed, giving spectral and spatial information. For the initial spectra elaboration, the PCA was conducted on pre-processed data. Figure 5 shows the distribution of the score plot in the PCA of spectra from the berries categorized into two classes and four classes. This unsupervised exploratory analysis demonstrates distinguishable variations in the spectra pertaining to the existence and categories of defects. Figure 5A depicts the distribution of sound and damaged fruit, which can be seen as sound fruit is distributed mainly in quadrant 1, while damaged fruit is mostly in quadrants 2 to 4. The overlapped sound and damaged fruit in quadrant 1 are due to the mild damaged fruit, which has a similar characteristic to sound. This overlapped similarity behavior is also observed in four classes of PCA plots depicted in Figure 5B.

The performance of PLSDA classification is summarized in Table 1. The selected model exhibited a sensitivity of 96.1 ± 2.1% for the “sound” category and 93.7 ± 0.6% for the “defective” category. Because of the inherent symmetry in the two-class discriminant problem, the specificities for “sound” were 93.7 ± 0.6%, and for “defective” they were 96.1 ± 2.1%. This resulted in an overall classification accuracy of 93.9 ± 0.6% and a mean classification error rate of 5.1 ± 1.1%.

**Figure 6:**
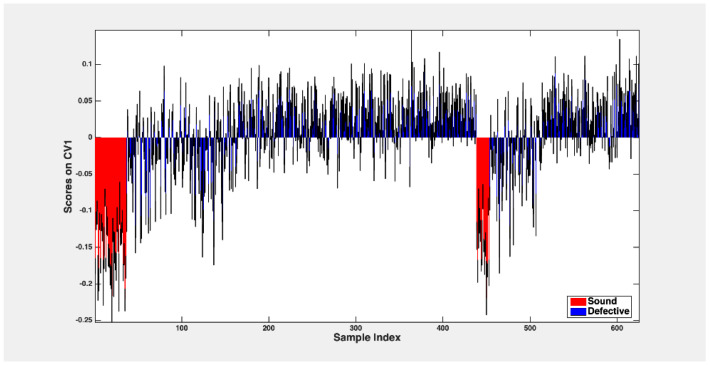
Mean scores and confidence intervals were assigned to samples from two classes (sound and defective fruit).

These observations are graphically depicted in Figure 6, illustrating the outer loop sample’s mean scores across the PLS-DA model’s singular canonical variate. The data are averaged over 50 rDCV runs, including 95% confidence intervals. The graphical representation shows that a significant portion of sound samples exhibits scores lower than −0.1, while scores higher than this threshold are assigned to defective fruit.

These findings attest to the high accuracy and robustness of the model in identifying defective fruit, being obtained with a method (rDVC) securing a substantial degree of independence from the subset on the training sample set. The repeated double cross-validation methodology provides both a precise estimation of the classification accuracy on test samples and generates confidence intervals, offering valuable insights into the reliability of the results. This robustness remains intact, even taking into account the variable selection procedure incorporated into the approach. With every unique model calculated for every outer loop split, individuating a specific set of variables, the approach facilitates the assessment of the robustness of the identified predictors.

**Figure 7:**
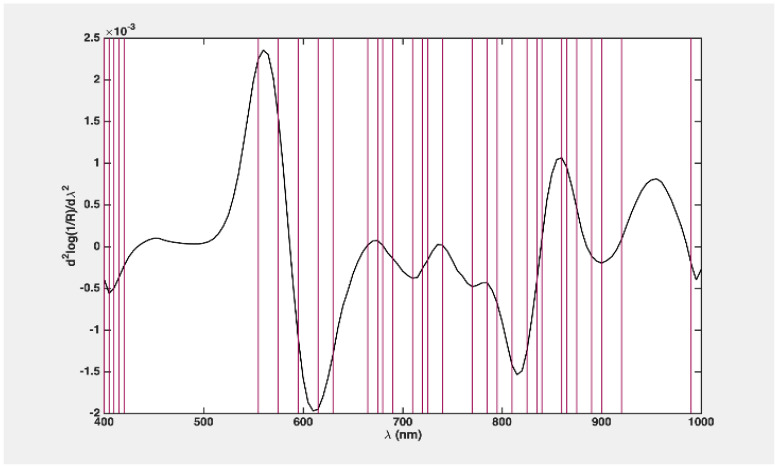
Variable loading plot for two classes of problem classification (red lines in the graph indicate the wavelengths used for the PLS-DA model).

In this study, 34 variables were selected, as illustrated in Figure 7. The refined model demonstrated enhanced classification accuracy (Table 2), achieving an accuracy of 94.9 ± 0.4. The sensitivity for Class 1 (and specificity for Class 2) experienced notable improvement, reaching 99.3 ± 1.1.

**Figure 8:**
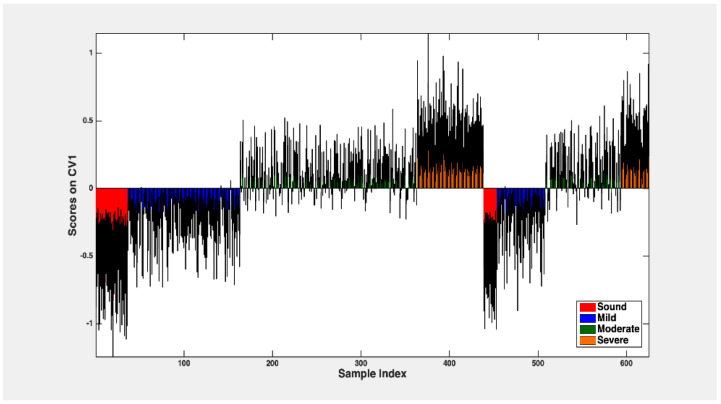
Mean scores and confidence intervals assigned to samples from four classes (sound, mild, moderate, and severe damaged fruit).

In the case of the four classes classification, the rDCV strategy was employed to confirm both the results of CovSel and PLSDA, with mean scores and confidence intervals illustrated in Figure 8. As delineated in Table 3, with a specific emphasis on the classification efficacy pertaining to the samples within the outer loop, the model demonstrated a sensitivity of 86.6 ± 2.3% and a specificity of 95.3 ± 0.6% for the “sound” fruit class. Additionally, the model exhibited a sensitivity of 70.2 ± 2.3% and a specificity of 88.6 ± 0.8% for the “mild defective” class, a sensitivity of 71.9 ± 1.7% and a specificity of 86.6 ± 0.9% for the “moderate defective” class, and a sensitivity of 83.1 ± 1.7% and a specificity of 93.1 ± 0.7% for the “severe defective” class. As expected, when looking at the distribution of wrong predictions, they encompass “neighboring” severity grades.

**Figure 9:**
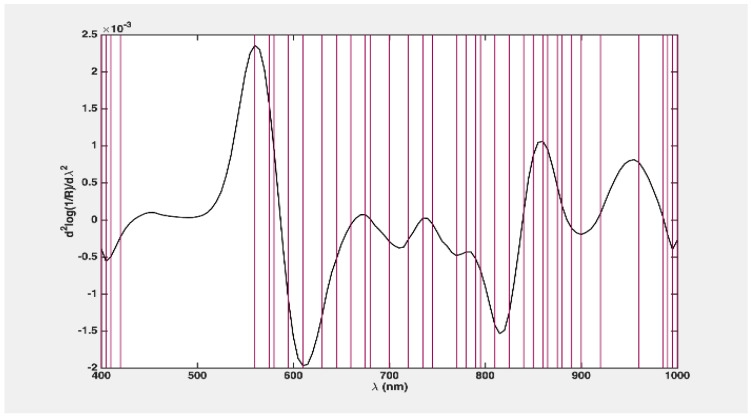
Variable loading plot for four classes of problem classification (red lines in the graph indicate the wavelengths used for the PLS-DA model).

As previously highlighted for the two-class problem, the prediction model based on four classes demonstrated high consistency and less reliance on the particular subset of training samples used. Correspondingly, as for the selection of the variable phase, integrated into the discrimination strategy, a consistent subset of 34 variables (depicted in Figure 9) was chosen in over 80% of cases. Specifically, these variables were selected in at least 400 out of the 500 calculated models from 10 outer loop splits and involving 50 time runs. To further evaluate the effectiveness of this selection, a PLS-DA model was developed using exclusively these 34 variables and subsequently validated through the same rDCV strategy, the result is demonstrated in Table 4.

**Figure 10:**
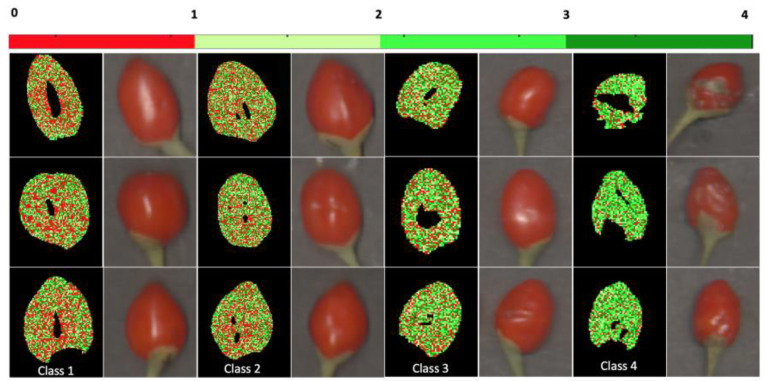
Pixel-by pixel classification of fruit into four classes: sound goji berry (Class 1), mildly damaged (class 2), moderately damaged (Class 3), and severely damaged fruit (Class 4).

As observed for the two-class problem, also, in this case, accuracy improved for the sensitivity of the class sounds (from 86.6 to 90.1), and generally for all the classes, as well as for the specificity, which increased by about 1% in all cases, contributing to an accuracy of 75.7%. Overall, the results of this study allowed an excellent ability of the model to detect and discriminate defective fruit from sound ones, which is the main objective of the optical sorting operation. Less acceptable accuracy is reported, particularly for sensitivity when four classes are considered but with a higher error between the two central classes, mild and moderate, as shown in the confusion matrix in Table 5. These results can be expected since errors in discrimination increase with the number of classes [32,33]. Figure 10 expresses the visual classification of the four classes of problems, used classifying pixel-by-pixel. The prediction image reflects the output of this classification, showcasing how, with the decrease of pixels classified as sound (red color), fruits are classified as mild, moderate, or severe damage (green color).

It is worth mentioning that the goji berry is a perishable fruit. Sound goji berries should be stored separately from damaged fruit. This is important because damaged fruit can harbor pathogens or degrade faster, potentially causing spoilage. This can occur by the cross-contamination of fruit occurring either by physical contact or through the moisture released by decaying fruit, which creates a favorable environment for the growth of fungi and bacteria. Thus, the early removal of infected fruit is critical to ensure high quality fruit, extend shelf life, and reduce waste. This is the first study aiming for the early detection and discrimination of defective fruit. Moreover, this work demonstrates high reliability, through validation with rDCV, which with a nested approach, repeated the model calibration for 500 times. A limited number of the cited papers incorporate the rDCV procedure. For example, [33] affirmed that a hyperspectral imaging system operating over the visible and near infrared ranges yielded promising outcomes for early disease recognition in blueberries by applying PLS-DA. In this case, for the two classes (i.e., 200 healthy and 200 diseased fruit), an accuracy of around 0.94 for the calibration and 0.9 in the CV was obtained by using several spectral pre-processing methods, autoscale, Log (1/R), and variable selection from 685–1000 nm. Another study reported the classification of bruised from sound kiwi fruits by using 1024 wavelengths ranging from 408 to 1117 nm, combined with a parallelepiped classifier, obtained an accuracy of 95.5% [20]. Additionally, the classification of 60 “Lingwu” long jujube fruits with internal bruises was reported by applying a hyperspectral image combined with a PLS-DA classifier in the visible and near-infrared ranges [22]. They obtained accuracy in calibration and prediction of 85.56 and 92.22%, respectively, achieved through the application of detrending pre-processing to the spectra. The classification of skin damaged from sound mango fruits by using 13 selected wavelengths was also reported, of which seven of them were also used in this study (i.e., 700, 730, 750, 770, 780, 890, and 900 nm), obtaining an accuracy of 91.4% [21]. Furthermore, HSI coupled with PCA to classify 70 sounds out of 27 citruses with defective skin was reported, reaching an accuracy of 97% by using two wavelengths, 680 and 715 nm, and also included among the 34 variables for goji [34]. HSI coupled with ANN successfully classified 90 sounds from 225 defective achacha fruit with an accuracy of 97% by using wavelengths which were also used in this paper at (570, 680, 710, 750, 820, 930, and 970 nm) [35]. These specific wavelengths potentially elucidate the physiological changes, including biochemical and structural transformations, that occur in goji berries during their maturation and storage. The spectral range from 400 to 680 nm is correlated with carotenoids, recognized for imparting the red coloration observed in goji berries. Zeaxanthin has been identified as the predominant pigment in this context, which changes by storage [36,37,38]. Concurrently, the spectral range from 715 to 750 nm has been linked to variations in water content within the berries [39]. Defective berries may have different water content due to spoilage or cellular damage, leading to variations in spectral reflectance. Therefore, 820, 930, and 970 nm wavelengths exhibit associations with sugar content in goji berries [38]. Defective goji berries may have reduced sugar content and altered sugar composition due to damage or stress. Additionally, Ref. [40] successfully classified the early development of green mold in citrus fruit by using only five wavelengths of 650, 660, 700, 750, and 760, of which three were applied in this paper (i.e., 650, 700, and 750 nm). This study achieved the optimal classification model by focusing on two classes, namely “sound” and “defective.” This strategic choice resulted in a notably high prediction accuracy, reaching an overall accuracy of 94.9% with a minimal 3.1% error. Noteworthy is the limited number of misclassifications, with only two sound fruits incorrectly classified as defective and 18 defective fruits misclassified as sound, over 574 fruits. Thus, such a high level of accuracy demonstrates that the system could effectively handle real-time sorting with minimal error, a critical requirement for online applications. Although some misclassifications occurred, the low numbers suggest the system is highly reliable to be scaled up for online sorting. The results, therefore, support the feasibility of using this method in an operational environment where rapid and accurate sorting is essential. The observed prediction errors in both the two-class and four-class defect models can be attributed to the challenges in predicting defects when still at the developmental stage. Although a satisfactory model was achieved, further study is needed to build a more robust classification model by adding a data set of goji berries from different seasons, cultivation regions, and agricultural practices.

## 4. Conclusions

The results of this study demonstrate the significant potential of hyperspectral imaging for the early detection of defects in goji berries. Using few spectral variables, the method provided a consistent and reliable classification of sound and defective fruit. The combination of hyperspectral imaging and the Partial Least Squares Discriminant Analysis (PLS-DA) model, validated through repeated cross-validation (rDCV), achieved high performance with an accuracy of 94.9% for the two-class classification (sound and defective). The model showed a sensitivity of 99.3% for the “sound” class and 94.5% for the “defective” class, with a minimal % overall class error of 3.1%. In the more complex task of classifying four defect levels (sound, mild, moderate, and severe), the model also performed reliably, achieving an overall accuracy of 75.7%. Although the sensitivity for individual defect levels was lower (e.g., 71.5% for mild, 72.5% for moderate), the system still proved to be robust in categorizing various defect types. These results highlight the feasibility of implementing this approach for real-time optical sorting, improving the quality control of goji berries, extending shelf life, and reducing waste. Further research is suggested to strengthen the model by incorporating data from different seasons and growing regions.

## Figures and Tables

**Table 1:** Classification using PLS-DA for the two classes problem, as calculated in the outer loop during repeated double cross-validation (rDCV).

True Class	Sensitivity (%)	Specificity (%)	Accuracy %)	Error (%)
Sound	96.1 ± 2.1	93.7 ± 0.6	93.9 ± 0.6	5.1 ± 1.1
Defective	93.7 ± 0.6	96.1 ± 2.1		

**Table 2:** Classification using PLS-DA for the two classes problem, as calculated in the outer loop during repeated double cross-validation (rDCV) using 34 variables.

True Class	Sensitivity (%)	Specificity (%)	Accuracy (%)	Error (%)
Sound	99.3 ± 1.1	94.5 ± 0.4	94.9 ± 0.4	3.1 ± 0.7
Defective	94.5 ± 0.4	99.3 ± 1.1		

**Table 3:** Classification is performed using PLS-DA for the four-classes problem, as calculated in the outer loop during repeated double cross-validation (rDCV).

True Class	Sensitivity (%)	Specificity (%)	Accuracy (%)	Error (%)
Sound	86.6 ± 2.3	95.3 ± 0.6	74.5 ± 1.1	22.1 ± 1.1
Mild	70.2 ± 2.3	88.6 ± 0.8		
Moderate	71.9 ± 1.7	86.6 ± 0.9		
Severe	83.1 ± 1.7	93.1 ± 0.7		

**Table 4:** Classification using PLSDA for the four classes problem, as calculated in the outer loop during repeated double crossvalidation (rDCV) using 34 variables.

True Class	Sensitivity (%)	Specificity (%)	Accuracy (%)	Error (%)
Sound	90.1 ± 1.5	96.0 ± 0.4	75.7 ± 0.7	20.4 ± 0.7
Mild	71.5 ± 1.7	89.3 ± 0.6		
Moderate	72.5 ± 1.4	86.5 ± 0.9		
Severe	84.3 ± 1.7	93.1 ± 0.6		

**Table 5:** PLSDA confusion matrix for the four-class problem. The outer loop samples in rDCV, which serve as external validation samples, are used to calculate the percentages.

		Predicted Class (%)			
		Sound	Mild	Moderate	Severe
True Class	Sound	90.1 ± 1.5	9.9 ± 1.5	0.0 ± 0.0	0.0 ± 0.0
	Mild	12.4 ± 1.1	71.5 ± 1.7	16.0 ± 1.3	0.0 ± 0.0
	Moderate	0.1 ± 0.1	14.8 ± 0.9	72.4 ± 1.3	12.6 ± 0.9
	Severe	0.0 ± 0.0	0.1 ± 0.3	15.6 ± 1.7	84.3 ± 1.7

## Data Availability

The original contributions presented in the study are included in the article, further inquiries can be directed to the corresponding author.
